# A numerical study on capillary-evaporation behavior of porous wick in electronic cigarettes

**DOI:** 10.1038/s41598-021-89685-4

**Published:** 2021-05-14

**Authors:** Yihan Gao, Dian Li, Jiexiong Ru, Muyun Yang, Lehua Lu, Li Lu, Jinlu Wu, Zhonghui Huang, Yan Xie, Naiping Gao

**Affiliations:** 1 Shanghai New Tobacco Product Research Institute, Xiupu Road 3733, Shanghai, 201315 China; 2 China Tobacco Guangxi Industrial Co., Ltd., Beihu South Road 28, Nanning, 530001 China; 3 School of Mechanical Engineering, Tongji University, Caoan Highway 4800, Shanghai, 201804 China

**Keywords:** Applied mathematics, Computational methods

## Abstract

A mathematical model based on heat and mass transfer processes in the porous wick of electronic cigarettes was established to describe the atomization of e-liquids according to max liquid temperature, vaporization rate and thermal efficiency in a single puff. Dominant capillary-evaporation effects were defined in the model to account for the effects of electrical power, e-liquid composition and porosity of the wick material on atomization and energy transmission processes. Liquid temperature, vaporization rate, and thermal efficiency were predicted using the mathematical model in 64 groups, varying with electrical power, e-liquid composition and wick porosity. Experimental studies were carried out using a scaled-model test bench to validate the model’s prediction. A higher PG/VG ratio in the e-liquid promoted energy transfer for vaporization, and the e-liquid temperature was comparatively reduced at a relatively high power, which was helpful to avoid atomizer overheating. Compared with the other factors, wick porosity affected the thermal efficiency more significantly. The vaporization rate increased with a higher wick porosity in a certain range. The modelling results suggested that a greater wick porosity and a higher PG ratio in e-liquids helped to improve the overall thermal efficiency.

## Introduction

In recent years, health impacts of electronic cigarettes have attracted research attention^1,2^. An electronic cigarette mainly consists of an atomizer (porous wick with heating coils) to produce a stream of aerosol, a cartridge for liquid storage and a rechargeable battery. Often classified as tobacco products by regulations, electronic cigarettes work by vaporizing their liquid solutions (e-liquids), mostly consisting of propylene glycol (PG) and vegetable glycerin (VG) to create a nicotine aerosol (sometimes called vapor) which users could inhale^3–5^. Capillary action transfers the liquid and vaporization releases the vapor upon heating by the coil. Capillary-evaporation therefore refers to the physical phenomenon that the e-liquid is transported from the cartridge by capillary force and continues to supply the wick during heating.

Most literature on electronic cigarettes have studied their aerosol chemistry and toxicant emission profiles. Some studies qualitatively and quantitatively evaluate the impacts of single or multiple device and liquid variables, such as electrical power, liquid composition, and puffing topography^6–8^ on the aerosol released^9–11^. Others have measured the working temperatures of wicks and heating coils, and their effects on the aerosol chemistry and particle size distribution^12^. Generally, less studies are dedicated to make deeper understanding on the thermophysics in the system, such as the heat and mass transfer mechanisms behind the capillary-evaporation phenomenon. A group introduced the nicotine flux to describe nicotine inhalation dose and rate^13^, and by modelling and experimental approaches the group has illustrated the importance that thermophysics plays in understanding the aerosol delivery from electronic cigarettes^14^. A study found that liquid vaporization could account for nearly 95% of aerosol generation by using NaCl-dissolved liquids as seeds or markers, and built an unsteady-state lumped model to study the influence of puffing topography and e-liquid composition on nicotine emissions^15^. This model was based on a simplified assumption that the liquid transportation by wicking is sufficiently fast, without restricting the capillary-evaporation effects during vaporization. On wicking behaviors, literature on porous media for mass and heat transfer could provide useful references. For example, the so-called Leverett function describes the capillary pressure under liquid saturation^15^. Previous experimental data could be summarized to yield an empirical formula^16^. A one-dimensional flow of water in an unsaturated porous media was also studied via numerical simulation^17^.

Building on these previous studies, here we describe a mathematical model of the capillary-evaporation effects to predict the e-liquid atomization process for the low-power electronic cigarette device with the electrical power not more than 10 W. The main structural feature of this type of electronic cigarettes is that the heating coils wind around the outside surface of the porous wick. The model incorporated liquid temperature, liquid vaporization rate and thermal efficiency of electronic cigarettes under various operating conditions. Simulation outcomes were compared with experimental results using an expanded scale electronic cigarette. Using the mathematical model, the impacts of electrical power, e-liquid composition and wick porosity on the atomization were systematically explored, and the insights could help to optimize the design of electronic cigarettes to minimize over-heating of e-liquids leading to “dry puff” caused toxicant generation.

## Results

### Comparison between model predictions and experiments

The liquid temperature, vaporization rate and thermal efficiency were selected as the evaluation parameters during e-liquid atomization. The temperature here refers to the maximum temperature of the e-liquid during a puff. The vaporization rate denotes the ratio of the vapor mass to the puff duration during a single puff. The thermal efficiency is defined as the ratio of the sum of the heat of evaporation and latent heat to the total input energy of the coil during a single puff. Mathematical modelling were based on energy and mass conservation law to predict these parameters under given conditions. In order to verify the model, an experimental platform was built based on the similarity theory^18^.

**Figure 1:**
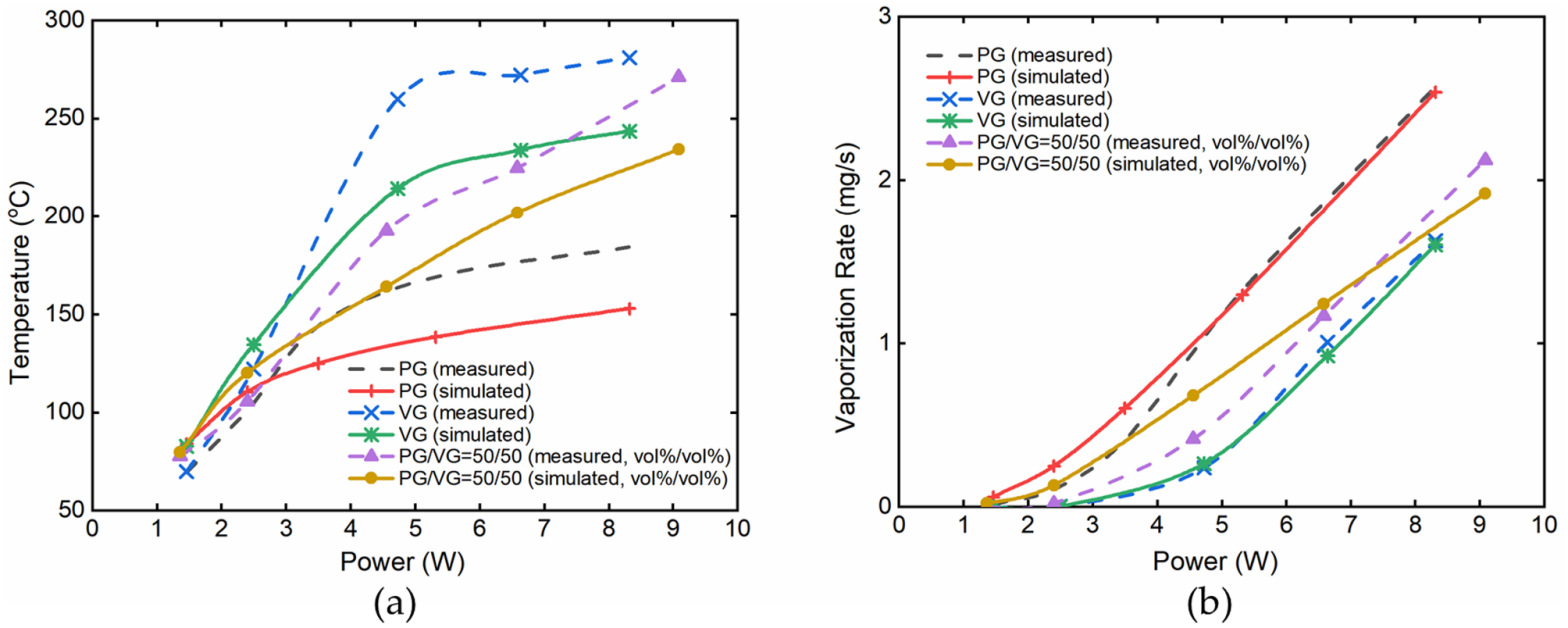
Correlations between applied electrical power and (**a**) liquid temperature, (**b**) vaporization rate at the condition of 100% PG, 100%VG, and PG/VG = 50/50 (vol%/vol%), 0.44 porosity. Puffing regime used: 3 s puff duration, 30 s puff interval and 55 mL puff volume, a square-wave puffing flow.

Figure 1 presents the comparison between the model predictions and the measured values of the liquid temperature (Fig. 1a), and vaporization rate (Fig. 1b) for the three liquid compositions. Within the electrical power range of 1.46–8.32 W, the measured liquid temperature increased with increasing power, but the maximum temperature did not exceed the corresponding boiling points of the liquids (about 188 °C for PG, 290 °C for VG, and 210 °C for 50/50 vol%/vol% PG/VG at atmospheric pressure^19^, respectively). Before reaching the e-liquid boiling point, the liquid temperature showed an approximately linearly increasing trend when the power applied was under ca. 5 W. The measured vaporization rates also increased with the rising electrical power in the same range. Among the PG, VG only and their binary combination solutions, the vaporization rate of PG was the highest. The model predictions of the liquid temperature and vaporization rate followed the similar trends and orders with those of the measurements. The discrepancies of the simulated and measured temperatures in Fig. 1a was because the simulated temperature is actually the average temperature of the porous wick with e-liquids inside due to the lumped parameter method used in the main part of the model, while the measured temperature is the surface temperature of the porous wick, which is near the heat source and thus rises faster than the internal temperature as well as the average temperature of the porous wick. The viscosity effect of the e-liquid atomization in the heated section of the porous wick and the similar effect of the vapor in the aerosol tube were not considered in the model. In fact, some of the e-liquids might remain in the pores of the porous wick and some of the vapor might be attached to the wall of the aerosol tube, which could explain that the calculated vaporization rates were higher than the measured values in low power range in Fig. 1b.

### Liquid temperature

**Figure 2:**
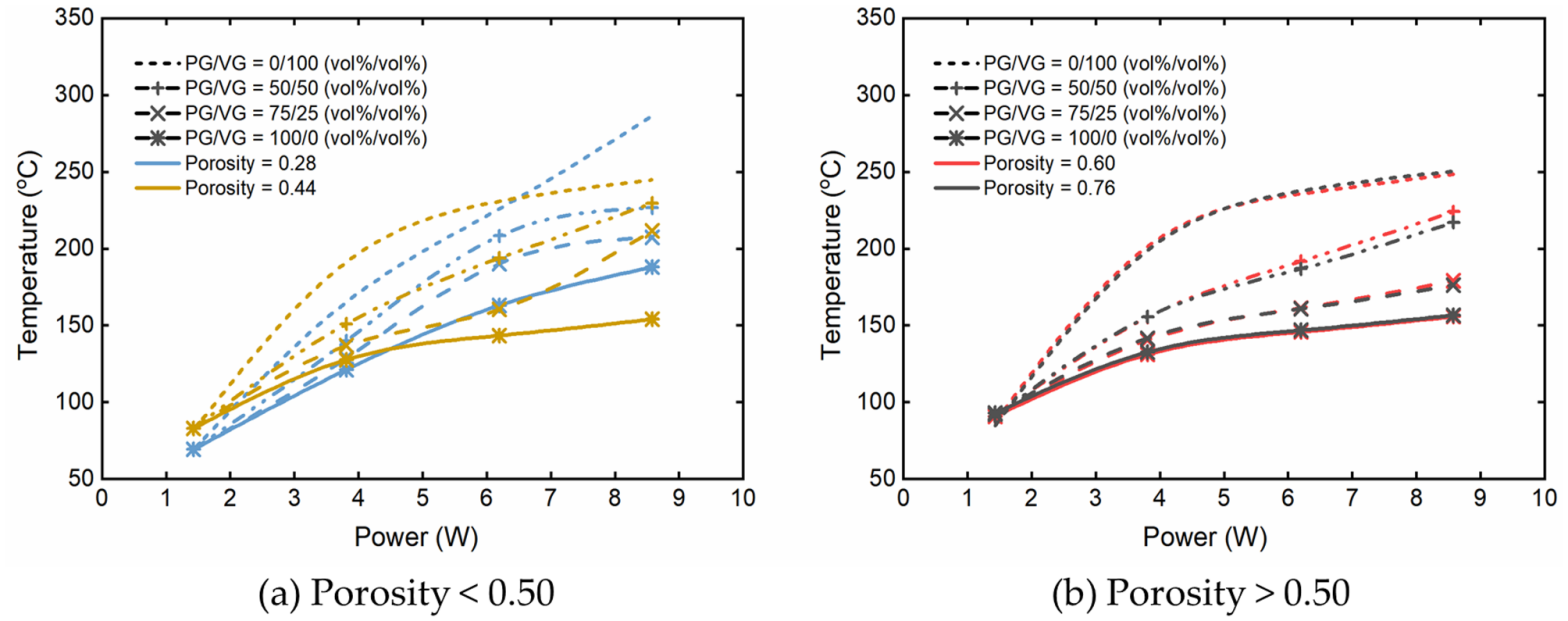
Model predictions of liquid temperature based on the input power for different liquids (PG/VG = 0/100, 50/50, 75/25, 100/0 vol%/vol%) at (**a**) porosity < 0.50 (0.28, 0.44) and (**b**) porosity > 0.50 (0.60, 0.76) with 3 s puff duration, 30 s puff interval and 55 mL puff volume.

Figure 2 shows the correlations between the applied power and the liquid temperature obtained by the model prediction, for different wick porosities and e-liquid compositions. For a given wick porosity, the predicted liquid temperature increased with the volume fraction of VG in e-liquids when the power was above 2 W. The results showed that the wick porosity was able to influence the liquid temperature more significantly than the PG/VG binary composition of the e-liquid. This trend could be subdivided into three separate relationships. At the lower power range (below 4–7 W, the exact power boundary depended on the liquid compositions) and at the porosity below 0.50, the liquid temperature increased with the increasing porosity. The opposite was observed at the high power (> 7 W) and the porosity below 0.50. When the porosity was above 0.50, the liquid temperature was relatively stable irrespective of the power applied.

### Vaporization rate

**Figure 3:**
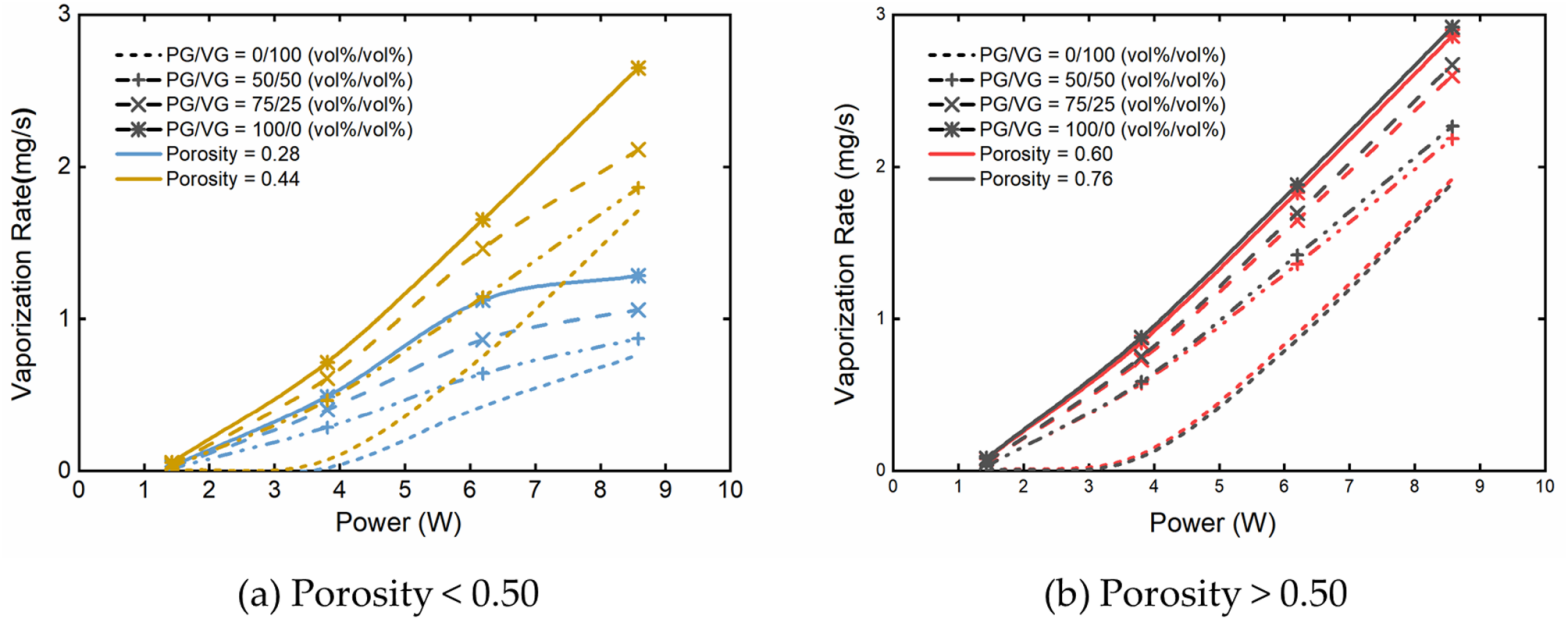
Model predictions of liquid vaporization rate based on the input power for different liquids (PG/VG = 0/100, 50/50, 75/25, 100/0 vol%/vol%) at (**a**) porosity < 0.50 (0.28, 0.44) and (**b**) porosity > 0.50 (0.60, 0.76) with 3 s puff duration, 30 s puff interval and 55 mL puff volume.

Figure 3 shows the correlations between the applied power and the vaporization rate predicted by the model. At a given wick porosity, the predicted vaporization rate was found to increase with the PG volume fraction in the e-liquid when the power was greater than 1.43 W. The vaporization rate also increased with the porosity (below 0.50) and with increasing power. However, the vaporization rate was basically unchanged when the porosity was above 0.50. In addition, the vaporization rate seemed to approach the maximum when the power was above 6 W and at a low porosity around 0.28.

### Thermal efficiency

**Figure 4:**
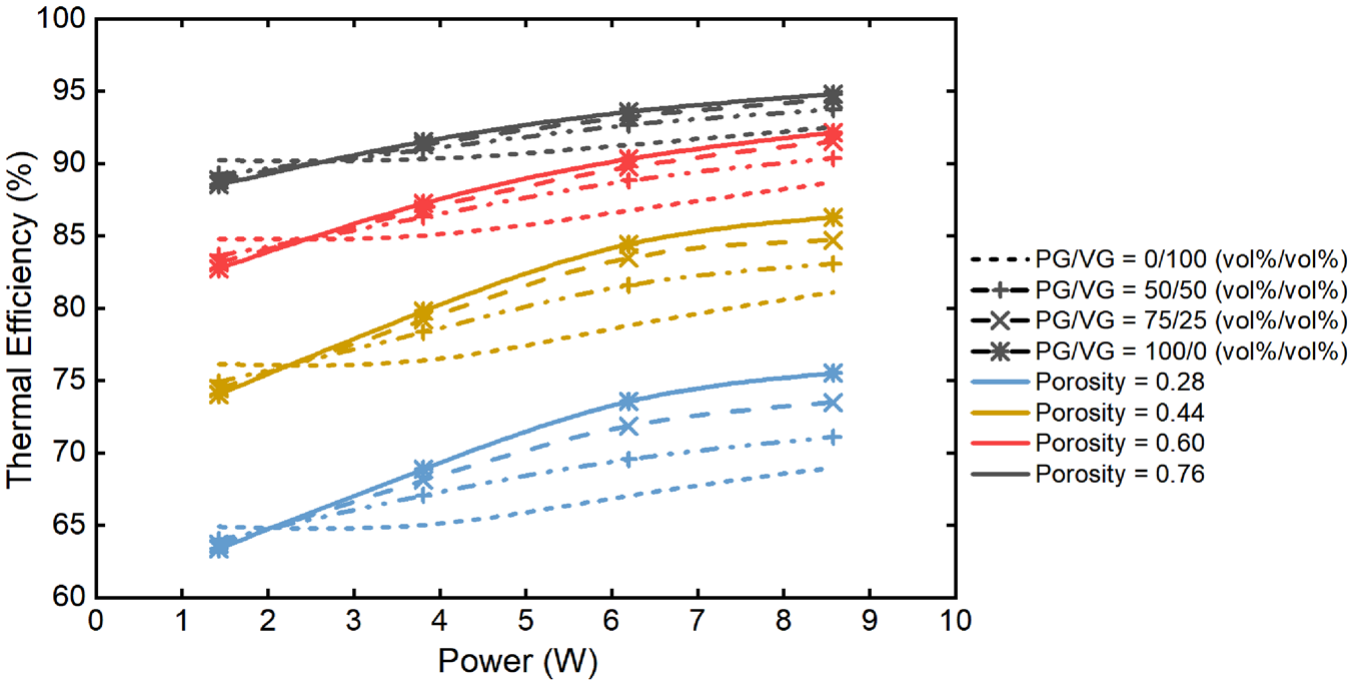
Model predictions of thermal efficiency based on the input power for different liquids (PG/VG = 0/100, 50/50, 75/25, 100/0 vol%/vol%) at 0.28, 0.44, 0.60, 0.76 wick porosity with 3 s puff duration, 30 s puff interval and 55 mL puff volume.

Figure 4 shows the calculated thermal efficiency by the model. It can be seen that the thermal efficiency increased with the VG volume fraction in the e-liquid at low power (below 2 W); while it increased with the PG volume fraction at a higher power. The thermal efficiency increased with the increasing wick porosity across all the conditions. This figure also shows the hierarchical distribution of the thermal efficiency according the wick porosity.

### Energy conversion

**Figure 5:**
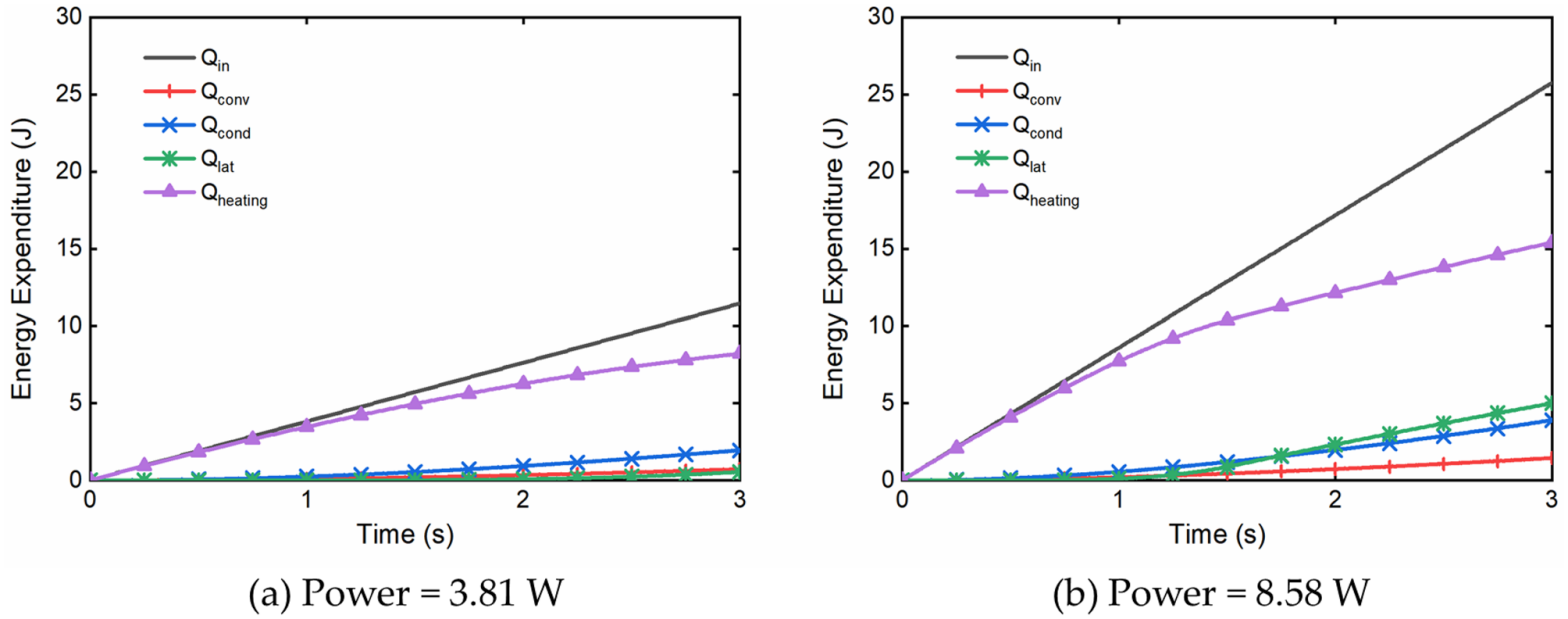
Predicted energy expenditure for a 50/50 (vol%/vol%) PG/VG liquid at two power levels: (**a**) 3.81 W and (**b**) 8.58 W, with 0.44 wick porosity, 3 s puff duration, 30 s puff interval and 55 mL puff volume. *Q*_*in*_ is the input electrical energy; *Q*_*conv*_ is the convective energy loss due to the puffing air; *Q*_*cond*_ is the conductive energy loss to the surrounding; *Q*_*lat*_ is the energy used for liquid boiling and evaporation; *Q*_*heating*_ is the energy used for heating the wick and the liquid within.

The numerical model could also be used to study the energy conversion during a single puff. An example of such calculations at a low and high powers are shown in Fig. 5. All energy expenditures were found to increase with the power from 3.81 to 8.58 W. Most of the input energy are used for heating the wick and liquids and switched from fast to slow with the puffing time. The proportion of the latent heat in the total input energy was small, but apparently increased with power. The heat losses due to the convection and conduction appeared always much smaller percentage of the input energy. As the puffing time reached 1.25 s (Fig. 5b), an inflection point on the curves could be seen, and the proportion of the energy used for heating the wick and liquids out of the input energy began to decrease, especially during the late stage of the puff. This was because e-liquids started boiling at this point.

## Discussion

Based on the thermophysics of a generic e-cigarette aerosolization process, a model of the heat and e-liquid mass transfer processes in the porous wick, driven by the capillary-evaporation effect, was established in this work. Both composition, and wick porosity on the e-liquid atomization process, especially on three key parameters governing an e-cigarette aerosolization: the liquid temperature, e-liquid vaporization rate and the thermal efficiency. Experimental validation of the overall approach was carried out by using an expanded-scale e-cigarette test platform. The experimental and numerical simulated outcomes matched reasonably (Fig. 1).

### Effects of electrical power on atomization

The results in Figs. 3, 4, 5 show that the liquid temperature, liquid vaporization rate, and the thermal efficiency of atomization all increased with the rising electrical power during a puff. The capillary-evaporation effect could be observed at an electrical power above ca. 6 W with a wick porosity about 0.28 (Fig. 3). It shows that a liquid vaporization rate could be limited when the maximum liquid mass transfer rate was reached by the capillary transport, and the performance of atomization thus restricted at a higher power with a lower wick porosity. This could result in overheating of any residual liquid within the wick and cause them to suffer excessive thermal decomposition.

### Effects of e-liquid composition on atomization

In both the experimental and numerical studies, the boiling temperature, the vaporization rate of the binary PG/VG e-liquid and the thermal efficiency changed with the PG/VG ratio, which is the result of the differentiated volatilities of the two main liquid constituents and their non-azeotropic mixtures. With VG (b.p. ~ 290 °C) and PG (b.p. ~ 188 °C) at the ambient pressure, a higher VG ratio in the binary e-liquid will result in a higher boiling temperature of the liquid system^19^, thus raising the upper temperature limit of the wick. A higher boiling temperature of the liquid also caused a greater degree of both convective and conductive heat losses, therefore consistent with the lower thermal efficiency. On the other hand, PG has a lower latent heat than that of VG, meaning that PG-rich liquids could vaporize more rapidly than VG-rich liquids at the same energy input. Therefore, the composition of the binary system can influence these the vaporization rate and the likelihood of dry wicking.

### Effects of wick porosity on atomization

The wick porosity directly affects the amount of e-liquid hold within the wick’s heated zone and it also influences the rate of capillary flow from the transportation zone. As the results shown here, these properties consequentially affected the atomization through corresponding changes in the effective heat capacity, the effective thermal conductivity, and the rate of evaporation and the total mass transfer of the porous wick.

When the wick porosity rises, the solid material in the wick decreases, increasing amount of e-liquid fills the pores. Depending on the wick material of cotton, the liquid components may have a higher heat capacity and lower density than the solid component in this study. This difference could increase the effective heat capacity and decrease the total mass of the wick, which may have contributed to the opposite trends of the temperature at either lower or higher power ranges.

With lower power inputs, before the temperature reached the boiling point of the e-liquid in the wick, the energy was mainly utilized to heat the combined wick-liquid system. The negative effect of the wick porosity on the total mass in this work would be greater than the positive effect on the effective heat capacity, which resulted in the increase of the temperature. On the other hand, the liquid temperature, as the most important factor, also influenced the partial pressure of the evaporating species. At lower power inputs, the vaporization process was mainly under the evaporation regime. According to the model, the vaporization rate was proportional to the partial pressure and was inversely proportional to the liquid temperature. The partial pressure of the evaporating species could be regarded as an increasing function of the temperature, which could be proved mathematically by taking the 1st derivative of the partial pressure with respect to the temperature. When the liquid temperature increased with the porosity, the growth of the partial pressure was much faster than the liquid temperature itself, resulting in an accelerated evaporation and hence the higher vaporization rate. However, there was a limitation for the wick porosity in controlling the vaporization rate. When the wick porosity was above 0.60, the two opposite effects of the porosity on the total wick-liquid mass and the effective heat capacity reached a similar degree, the temperature and the vaporization rate appeared to remain unchanged.

With higher power inputs, the liquid temperature rapidly approached the boiling temperature of the liquid and the vaporization process entered the boiling regime. The thermal energy contributed to not only the heating of the wick-liquid system, but also to the latent heat of the e-liquid to vaporize the liquid by boiling off. More input energy from the heating coil was consumed by overcoming the energy for heating the wick and liquids. Under this condition, an increased porosity could cause a decreasing effective thermal conductivity of the wick. In our test, the thermal conductivity of the liquids was lower than that of the wick skeleton. Although the temperature might increase with the porosity at relative low power as explained before, the positive impact of the decreasing effective thermal conductivity on the thermal efficiency was much stronger than the negative impact of the increasing temperature, which led to the reduction of the convective and conductive heat losses and thus increased the thermal efficiency. The apparent hierarchical distributions in Fig. 4 indicates the impact of the porosity was stronger than the power inputs and the e-liquid composition.

### Effects of e-liquid composition on the capillary transport

Compared with the other studies^14,15^, one of the most important uses of this model was to calculate the maximum capillary mass transfer rate. In this model, the pores within the wick were assumed to be the same shape and uniformly distributed. The more pores with a given wick dimension, the greater the capillary-led liquid transport would be. Therefore, the maximum mass transfer rate of the liquid would generally increase with the porosity, which supports a greater vaporization rate. The liquid vaporization rate could be conjectured to increase by enhancing the power inputs until it reached the maximum with a given porosity.

### Energy conversion during atomization

The model predicted that most of the input energy from the heating coil was used to heat the porous wick and the e-liquid before boiling, and thereafter to drive the liquid-to-vapor phase change after reaching the boiling point of the liquid. The inflection points in Fig. 5 displayed the transition from the evaporation to the boiling regime. While the total energy consumption increased with the increasing power input, the energy used for vaporizing the e-liquid and heating the system during a puff might be more sensitive to the change of electrical power than the convective heat loss and conductive heat loss, which led to the growth trend of thermal efficiency with the increasing power (Fig. 4). Therefore, for a given e-cigarette design, shortening the evaporation relative to the boiling of the e-liquid enhanced the aerosol yield.

### Limitations

In addition to the assumptions made in constructing the model, this study assumed a lumped-parameter approach to thermal vaporization process. Thus, the calculated liquid temperature and vaporization rates represented the average values of the heating section. The experimental temperature measuring locations were near the coil, which might have contributed to the simulated temperatures being lower than the measured values. In a real e-cigarette, the distributions of the heating temperature, e-liquid composition and other parameters are three-dimensional and varies according the way the e-cigarette is positioned. Any local non-equilibrium heat transfer processes could be considered in future refinement. The experimental e-liquids tested did not contain any nicotine, which prevented the evaluation of nicotine transfer in this work.

## Conclusions

A thermophysical model of aerosolization process was constructed and verified experimentally in this study. The electrical power, e-liquid composition, and wick porosity were found to influence the liquid temperature, liquid vaporization rate, and thermal efficiency of e-cigarettes. Greater power inputs promoted a higher liquid temperature, liquid vaporization rate and thermal efficiency at a constant wick porosity and e-liquid composition. A too high input power led to rapid the liquid temperature increases and caused dry wick (the run-off of coil temperature). Moderately high PG ratios in the e-liquid at a high power promoted the energy transfer for vaporization and reduced the evaporation at lower temperatures. However, the high PG ratio effect at a lower power traded off against the effect of reduced thermal efficiency. Wick porosity played a more important role in the thermal efficiency than that of the electrical power input and the e-liquid composition. Wick porosity determined the maximum of vaporization rate by controlling the effective thermal conductivity, while the effect for wick porosity above 0.60 was marginal in this study. Therefore, for higher thermal efficiency in the design of e-cigarettes, it is recommended to firstly increase the wick porosity and secondly increase the PG ratio in e-liquid at relative high powers.

## Methods

### Materials

Test e-liquids used in the verification experiments were prepared and composed of PG and VG according to the required volume ratio. Liquid PG and VG reagents were purchased from Aladdin (Shanghai, CN). Cambridge filter pads (44 mm diameter) were purchased from Whatman Corporation (USA) and used for trapping aerosol particulates. The heating coil of 0.5 mm diameter was made of nickel-chromium alloy. K-type thermocouples of 0.5 mm diameter were used for temperature monitoring.

### Mathematical modelling

**Figure 6:**
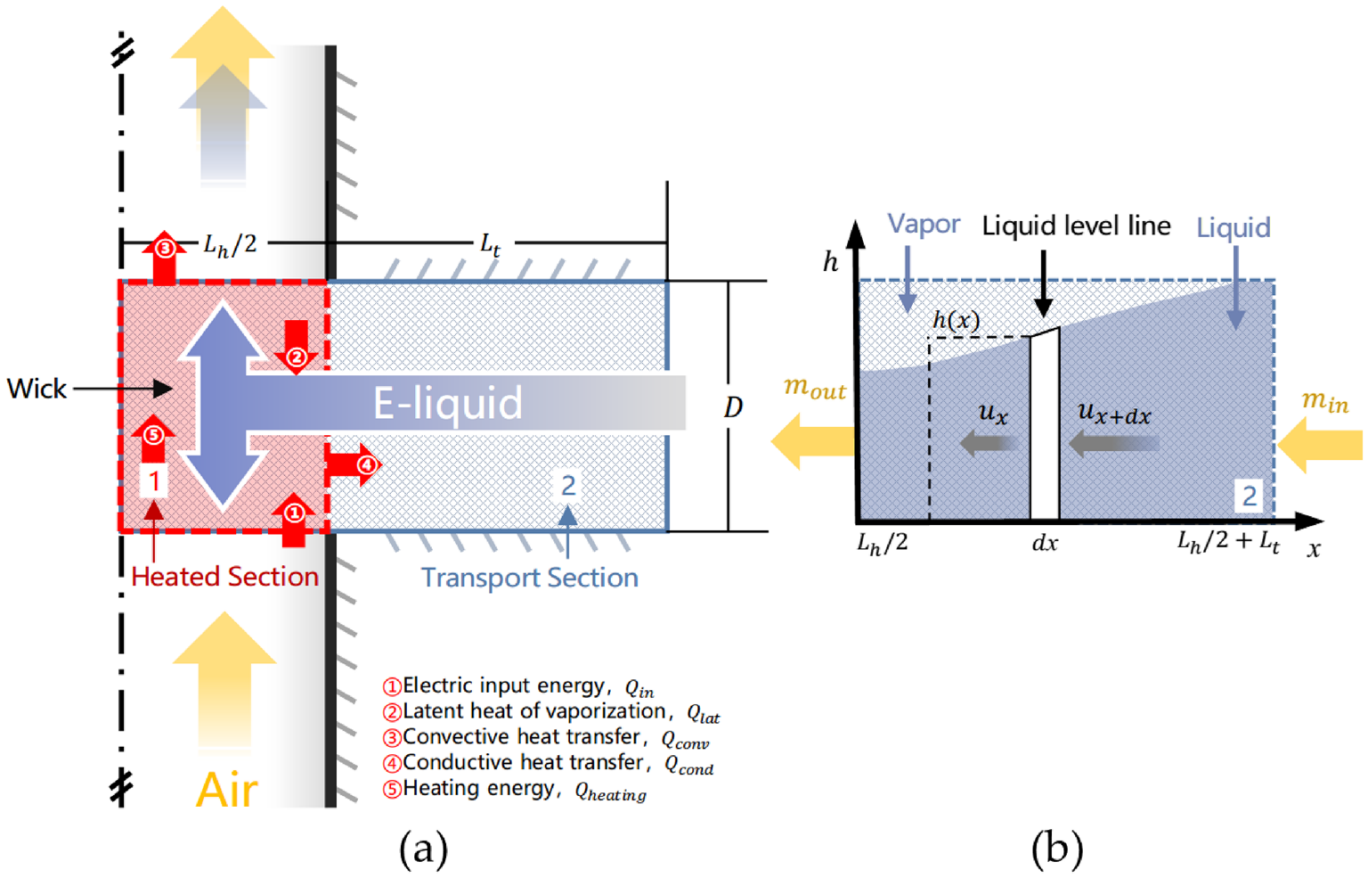
Schematic diagrams of (**a**) a horizontal porous wick with a heated section (zone 1), surrounded by its heating coil, and an e-liquid transport section (zone 2) conducting e-liquid from the liquid reservoir to the wick. Puffing air flows across the heated section and carries the vaporized liquid away as a heated vapor, which subsequently condenses into an aerosol stream; (**b**) a micro-element within the transport section of the wick. The liquid level line illustrates a transient boundary between the remaining liquid and the vapor at time t. h represents the liquid level within the wick.

To derive the model, the geometry of an e-cigarette’s vaporization zone was represented by a horizontal cylindrical wick and coil, which was intersected by a vertical cylindrical air passage (Fig. 6a). The wick was treated as incompressible porous media. It was divided into two sections, a heated section (zone 1, Fig. 6a) where the wick was surrounded externally by the heating coil, and a transport section (zone 2, Fig. 6a) which connected the wick and the e-liquid cartridge at ambient temperature during e-cigarette operation. When a puff is taken, the e-liquid contained in the heated section received the energy from the coil and evaporated rapidly to produce a stream of vapor, which was then carried out of the vaporization zone by the puffing air flow through the air passage.

In addition to the conditions described above, this model assumed: (1) The coil and e-liquid temperatures and the e-liquid composition were uniform; (2) Other physical properties were isotropic; (3) The wick, e-liquid and aerosol were a rigid porous medium, Newtonian fluid and ideal gas, respectively; (4) The local heat equilibrium (LTE) exists between the e-liquid and the wick; (5) The capillary flow of e-liquids in the transport section is regarded as axial flow, while the radial flow is ignored because of the geometry.

Assuming that energy conservation was maintained, the input electrical energy was consumed by four parts: the latent heat of e-liquid vaporization via evaporation and boiling, the energy used to heat the wick with the e-liquid, the heat loss to the flowing air by convection during a puff and to the device surrounding by conduction. Each of the energy terms could be described and computed by using an unsteady energy conservation equation:

1 $$ \dot{Q}_{input} = \dot{Q}_{lat} + \dot{Q}_{conv} + \dot{Q}_{cond} + \dot{Q}_{heating} , $$

where $$\dot{Q}_{input}$$ is the input power (W); $$\dot{Q}_{conv} = h_{a} A_{s} \left( {T - T_{0} } \right)$$ is the convective heat transfer due to the puffing air (W), where $$h_{a} $$ is the heat transfer coefficient of a cylinder in cross flow $$\left( {{\text{W m}}^{ - 2} {\text{ K}}^{ - 1} } \right)$$, $$T$$ is the liquid temperature, $$T_{0}$$ is the ambient air temperature ($${\text{K}}$$), $$A_{s} = \pi DL_{h}$$ is the surface area of the heated section ($${\text{m}}^{2}$$), in which $$L_{h}$$ is the length of the heated section of the wick (m). $$\dot{Q}_{cond} = 2kA_{in} \left( {T - T_{0} } \right)/L_{t}$$ is the heat transfer loss via conduction through the wick to the e-liquid cartridges, where $$L_{t}$$ is the length of the transport section of the wick (m), $$A_{in} = \pi D^{2} /4$$ is the end surface of the wick ($${\text{m}}^{2}$$), $$k = k_{s} \left( {1 - \varepsilon } \right) + k_{l} \varepsilon$$ is the effective thermal conductivity of the total wick ($${\text{W }}\,{\text{m}}^{ - 1} { }\,{\text{K}}^{ - 1}$$), where $$k_{s}$$ is the thermal conductivity of the wick, $$k_{l}$$ is taken as the mass-weighted average of thermal conductivity of the e-liquid. $$\dot{Q}_{heating} = c_{p} m\left( {T - T_{0} } \right)/t_{a}$$ is the energy expending rate for heating the total wick, where $$c_{p} = m_{s} c_{p,s} /m + \left( {1 - m_{s} /m} \right)c_{p,l}$$ is the effective heat capacity of the total wick ($${\text{J}}\,{\text{kg}}^{ - 1} \,{\text{ K}}^{ - 1}$$), $$c_{p,s}$$ is the heat capacity of the wick, $$c_{p,l}$$ is the heat capacity of the e-liquid. For a binary e-liquid of PG and VG, this is calculated as the mass-weighted average. $$m = m_{s} + m_{l} = \pi D^{2} L_{h} \rho /4$$ is the total mass of the fully wetted wick (kg), $$m_{s} = \pi D^{2} L_{h} \left( {1 - \varepsilon } \right)\rho_{s} /4$$ is the mass of the wick skeleton without the e-liquid, $$m_{l} = \pi D^{2} L_{h} \varepsilon \rho_{l} /4$$ is the mass of the e-liquid in the wick, $$\rho = \varepsilon \rho_{l} + \left( {1 - \varepsilon } \right)\rho_{s}$$ is the effective density of the total wick ($${\text{kg }}\,{\text{m}}^{ - 3}$$), and $$t_{a}$$ is the puff duration (s). $$\dot{Q}_{lat} = \dot{m}_{out} h_{fg}$$ is the latent heat associated with the phase change from the e-liquid to the corresponding vapor, where $${\dot{\text{m}}}_{{{\text{out}}}}$$ is the e-liquid vaporization rate ($${\text{kg }}\,{\text{s}}^{ - 1}$$), and $$h_{fg}$$ is the latent heat of vaporization ($${\text{J }}\,{\text{kg}}^{ - 1}$$).

During a puff, e-liquid vaporization could be divided into two steps: an evaporation step and a boiling step. Within the evaporation step, the e-liquid temperature was below the lowest boiling temperature of the e-liquid composition (for a PG/VG system), and the vaporization rate was computed by the convective mass transfer. As the puffing went on, more energy was delivered by the heating coil and the e-liquid temperature increased and eventually reached the corresponding boiling temperature, the vaporization rate was now governed by the energy conservation equation of boiling. The vaporization rate ($\dot{m}_{out,i}$) is thus given as:

2 $$ \dot{m}_{out,i} = \left\{ {\begin{array}{*{20}c} {\dot{m}_{e,i} (T_{l} < T_{b} )} \\ {\dot{m}_{b,i} \left( {T_{l} \ge T_{b} } \right),} \\ \end{array} } \right. $$

where $T_{l}$ is the e-liquid temperature (K), $T_{b}$ is the boiling temperature (K), $\dot{m}_{out,i}$ is the vaporization rate of the e-liquid species $i$, $\dot{m}_{b,i}$ is the boiling vaporization rate of the species $i$, which could be calculated by the energy conservation equation. If the liquid temperature is below the boiling temperature, $\dot{m}_{e,i}$, the evaporating vaporization rate of liquid species $i$ is given by a convective mass transfer:

3 $$ \dot{m}_{e,i} = h_{D,i} \Delta \rho_{g,i} A_{s} , $$

where $$h_{D,i} = \frac{{D_{i} }}{D}Sh_{I}$$ is the convective mass transfer coefficient of the evaporating species $$i$$ ($${\text{m s}}^{ - 1}$$), $$D_{i}$$ is the diffusion coefficient in the air ($${\text{m}}^{2} \,{\text{ s}}^{ - 1}$$), $$D $$ is the cylinder diameter of the wick ($${\text{m}}$$), $$Sh$$ is the Sherwood number of evaporating mass transfer. $$Sc_{i} = \frac{{\nu_{a} }}{{D_{i} }}$$ is the Schmidt number, and $$\nu_{a}$$ is the kinematic viscosity of air ($${\text{m}}^{2} {\text{ s}}^{ - 1}$$). The convective mass transfer corresponding formula $$Sh = f\left( {Re,Sc} \right)$$ has the same mathematical form as the convective heat transfer corresponding formula $$Nu = f\left( {Re,Pr} \right)$$ in this study. $$\Delta \rho_{g,i}$$ is the density difference between vapor species $$i$$ depositing on the surface of the wick and in the air flow ($${\text{kg m}}^{ - 3}$$), $$\rho_{l,i} = \frac{{P_{i} M_{i} }}{RT}$$ is the density of the species $$i$$ on the surface of the wick, $$M_{i}$$ is the molar mass of the evaporating species $$i$$ ($${\text{kg mol}}^{ - 1}$$),$$ R$$ is the universal gas constant ($${\text{J mol}}^{ - 1} {\text{ K}}^{ - 1}$$), $$T$$ is the evaporation temperature (K), and $$P_{i} = x_{i} P_{i}^{*}$$ is the partial pressure of the species $$i$$ ($${\text{Pa}}$$), where $$x_{i}$$ is the molar fraction in the e-liquid, and $$ P_{i}^{*}$$ is the saturated vapor pressure of the evaporating species $$i$$ (Pa), which is calculated by the Antoine equation:

4 $$ \lg \left( {P_{i}^{*} } \right) = A_{i} - \frac{{B_{i} }}{{T + C_{i} }}, $$

where $$A_{i}$$,$$ B_{i}$$,$$ C_{i}$$ are all empirical coefficients of the Antoine equation^20,21^.

During the operation of an e-cigarette, the boiling temperature of the binary e-liquid (PG and VG) changes with the variation in liquid composition. The boiling temperature corresponding to the molar fraction of species $i$ is given by the mixed-gas law:

5 $$ \sum P_{i} = \sum \left( {x_{i} \cdot P_{i}^{*} } \right) = P_{a} , $$

where $P_{a}$ is the atmospheric pressure. The mass fraction of species $i$ ($w_{i}$) could be computed by the species mass balance:

6 $$ \frac{{dw_{i} }}{dt} = \frac{{dm_{l,i} }}{{m_{l} dt}} = \frac{{\dot{m}_{in,i} - \dot{m}_{out,i} }}{{m_{l} }} = \frac{{w_{i,p} \dot{m}_{in} - w_{i,v} \dot{m}_{out} }}{{m_{l} }}, $$

where $m_{l,i}$ is the mass of liquid species $i$ in the wick (${\text{kg}}$), $dt$ is the time differential (${\text{s}}$). The starting of the puffing is defined as the time when the porous wick becomes fully wetted. $\dot{m}_{in,i} = {\dot{\text{m}}}_{{{\text{in}}}} w_{i,p}$ is the inflow e-liquid mass from the cartridge, and $\dot{m}_{out,i} = \dot{m}_{out} w_{i,v,}$ is the released vapor mass of the aerosol, where $\dot{m}_{in}$ is the total inflow liquid mass, $\dot{m}_{out}$ is the total vapor mass of the aerosol, $w_{i,p}$ is the mass fraction of species $i$ in the parent e-liquid in the cartridge, and $w_{i,v}$ is the mass fraction of species $i$ in the aerosol.

Based on the mass conservation, the total mass inflow rate is calculated as $\dot{m}_{in} = \sum \dot{m}_{in,i} = \dot{m}_{out} = \sum \dot{m}_{out,i}$. In this work, we assumed that irrespective of the vaporization step, the vaporization rate was always limited by the maximum mass transfer rate of the capillary wicking capacity:

7 $$ \dot{m}_{out} = min\left( {\sum \dot{m}_{out,i} ,\dot{m}_{c,max} } \right), $$

where the maximum mass rate of capillary transport,${{ \dot{\text{m}}}}_{{{\text{c}},{\text{max}}}}$, which is described as follows.

The maximum e-liquid mass transfer rate occurred in the transport section of the wick and driven by the capillary action. Figure 6b is a schematic diagram of the transport section. From a macro level, the e-liquid in pores of the wick could be regarded as a continuum. The continuity equation for the capillary flow in the porous wick could be described by microelement analysis:

8 $$ - u_{x} \pi h_{x}^{2} \varepsilon \rho_{l} = - u_{x + dx} \pi h_{x + dx}^{2} \varepsilon \rho_{l} , $$

where $h$ is the e-liquid level of the considered microelement of the liquid continuum as shown in Fig. 6b (${\text{m}}$), $u$ is the axial velocity of the capillary transport flow (${\text{m s}}^{ - 1}$), and the minus sign in this equation represents the opposite direction to the $x$ direction; and $\varepsilon$ is the porosity of the wick. To calculate $u$, the liquid saturation is introduced as the volume ratio of the liquid continuum to the pore volume. Driven by the capillary pressure, e-liquid flowed spontaneously from the cartridge to the heated section. By calculating the Reynolds number of the capillary flow in the porous wick, the capillary flow of e-liquids could be proved to be laminar flow with the max range of Reynolds number $1.9 \times 10^{ - 6} \sim 5.3 \times 10^{ - 5}$ under the max electrical power 8.32 W. Because the max range of Reynolds number is below the range of the critical Reynolds number in the porous media 0.1–130^22^, this process is then proved to be laminar and follow the Darcy Law. Thus, the axial velocity, $u$, can be written by the momentum equation:

9 $$ u = - \frac{K}{{\mu_{l} }}\frac{{dp_{l} }}{dx}, $$

where $K$ is the permeability of the wick (${\text{m s}}$), $\mu_{l}$ is the dynamic viscosity of the liquid (${\text{kg m}}^{ - 1} {\text{ s}}^{ - 1}$), and $p_{l}$ is the liquid pressure (${\text{Pa}}$) associated with the capillary pressure $P_{c}$ by $P_{c} = P_{g} - P_{l}$, where $P_{g}$ is the vapor pressure and remains constant at $P_{a}$. Based on the dimensionless Leverett function $J\left( s \right)$^23^, the capillary pressure could be calculated as $p_{c} = \sigma \sqrt {\varepsilon /K} J\left( s \right)$, where $\sigma$ is the surface tension coefficient ($\mathrm{N }{\mathrm{m}}^{-1}$). Udell’s empirical $J(s)$ formulation is used as $J(s)=1.417(1-s)-2.120(1-s{)}^{2}+1.263(1-s{)}^{3}$^16^. Then the momentum equation is given as $u = \left( {\sigma \sqrt {\varepsilon K} /\mu_{l} } \right)dJ\left( s \right)/dx$.

The left boundary condition (Fig. 1b) of $\dot{m}_{out}$ is linked to the vaporization rate and the right boundary condition $s\left( {L_{h} /2 + L_{t} } \right) = 1$ is given for the complete wetting ends of the transport section. If the vaporization rate increases, the e-liquid level in the wick will drop and the saturation at all points within the wick will decrease, which will cause a rapid rise in the wick temperature at given power setting—this results in overheating of the residual e-liquid and often lead to their excessive thermal decomposition, in other words, leading to the so-called “dry wick” phenomenon. To avoid this dry-out failure of the wick, the saturation point where the transport section connects with the heated section is defined as the empirical minimum value 5%, which has been verified by the model results.

### Numerical calculation

The equations derived from the model were solved simultaneously using MATLAB (Version R2018a, 64-bit). The time terms were solved by the forward Euler method and the space terms were solved with the second order central differencing scheme. In the solution procedure, the maximum mass transfer rate of the wick was solved first. The instantaneous temperature, liquid composition, vaporization rate and thermal efficiency under the restriction of the capillary-evaporation effects were calculated in discrete time steps. The initial liquid temperature was set to 10 °C. Time steps of 0.01 s and space steps of 0.1 mm were used. The independence of time steps and space steps were tested separately by comparing the calculated results at the steps of 0.001 s and 0.01 mm.

After the model verification, a total of 64 conditions were numerical simulated, combining four e-liquid compositions (PG/VG = 0/100, 50/50, 75/25, 100/0, vol%/vol%), four electrical powers (1.43, 3.81, 6.20, 8.58 W) and four apparent porosity of the wick (0.28, 0.44, 0.60, 0.76). The maximum of porosity was based on a porous ceramic typically used for wicking. Physical properties of the prototype e-cigarette and parameters of simulation studies are shown in the Supplementary Information. All the physical properties of the e-liquids and the porous wick were at the mean value of the average boiling temperature and the ambient temperature.

### Model validation

**Figure 7:**
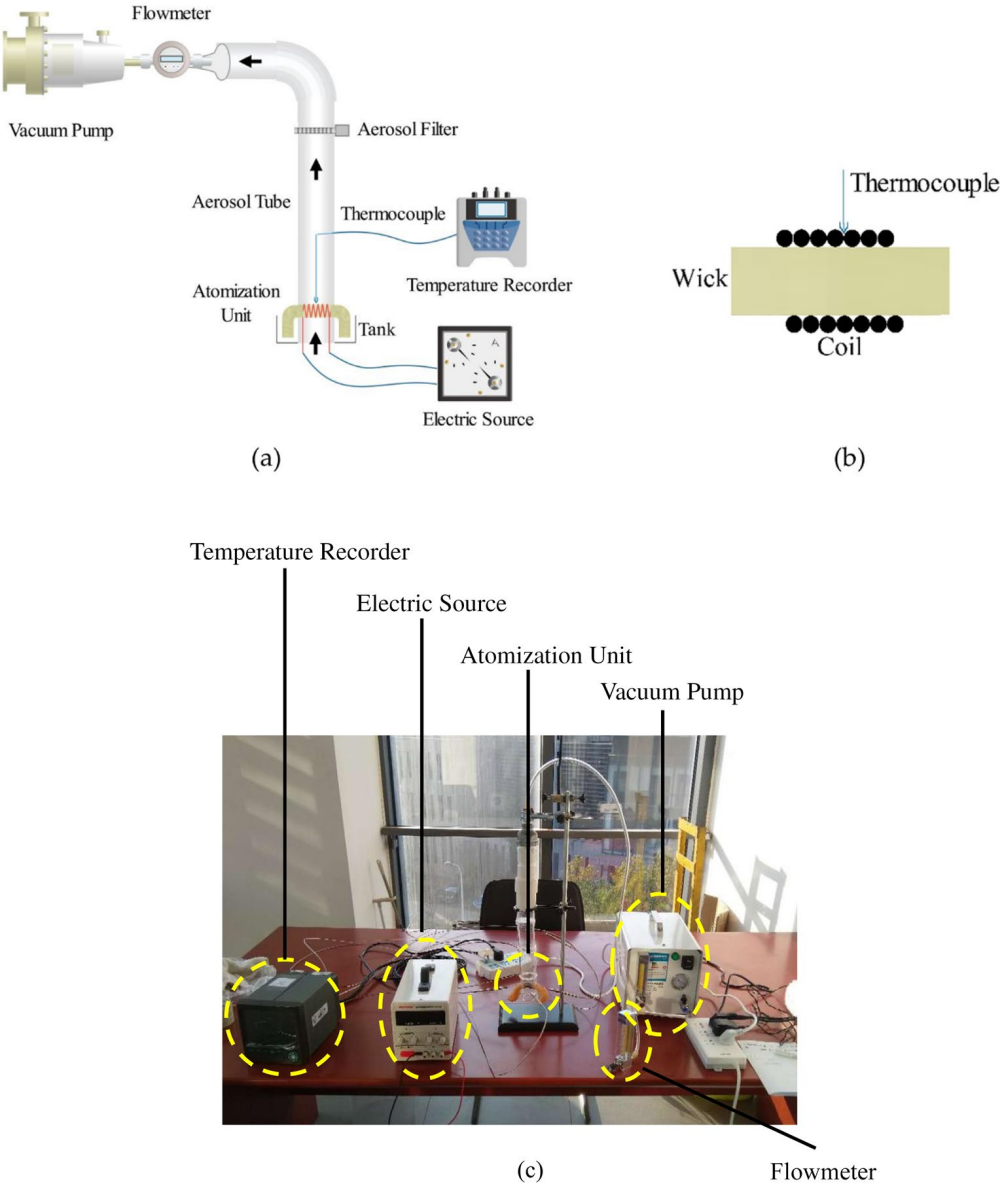
(**a**) Schematic diagram of experimental platform; (**b**) Detail view of coil and wick; (**c**) Physical picture of experimental platform (Note: The diameter and length of the prototype wick are 3 mm and 16 mm, respectively. The wick is made of cotton. The porosity of the wick is 0.44. The coil is wound on the outer surface of cylindrical wick. The measuring point of thermocouple is contacted with the coil. The diameter of the prototype aerosol tube is 8 mm).

Verification experiments were then performed using the expanded-scale experimental platform to reduce possible measuring errors that might be caused with a small-sized real e-cigarette, as shown in Fig. 7. The platform consisted of a vacuum pump to enact a puffing flow, an atomization unit to mimic a coil/wick system, and an electric source to supply a defined amount of energy to the heating coil. An air flowmeter, an aerosol filter (a 44 mm Cambridge filter pad) and a quartz aerosol tube, 0.5 mm diameter K-type thermocouples and a temperature recorder provided necessary flow and temperature sampling methods which were otherwise difficult to achieve within the confinement of a real-sized e-cigarette. The operational range of this set-up is given in Table 1.

**Table 1:** The main operational parameters of the scaled-up test platform.

Instrument	Working range	Precision
Temperature Recorder	− 200 ~ 1370 °C	± 3 °C (± 0.2% F·S)
Thermocouple	0 ~ 550 °C	± 2.5 °C (II Level Precision)
Electric source	0 ~ 30 V 0 ~ 10 A	± 0.5% (voltage) ± 0.1% (current)
Flowmeter	1 ~ 10 L/min	± 2.5%
Vacuum pump	0 ~ 30 L/min − 85 ~ 0 kPa	~

In order to ensure the experimental platform could be used to simulate the real e-cigarette, the dimensionless numbers of the experimental platform and real e-cigarette should be the same based on similarity theory^18^. For measurement convenience, the physical dimension of the expanded-scale model was set at 5 times of a prototype e-cigarette.

A total of 15 experimental conditions were tested as a combination of three liquid compositions (100% PG, 100% VG,PG/VG = 50/50 vol%/vol%) and five electrical powers (1.46, 2.40, 3.50, 5.32, 8.32 W for PG; 1.46, 2.50, 4.73, 6.64, 8.32 W for VG; 1.36, 2.40, 4.56, 6.58, 9.09 W for PG/VG = 50/50 vol%/vol%) which could be checked in the Supplementary Information. The wick porosity was measured as 0.44. All the e-liquids used in this study were prepared using the reagent grade of propylene glycol (PG) and vegetable glycerin (VG). The puff topography of the prototype e-cigarette followed the CRM81^24^ protocol: a square-wave puff flow curve, 55 mL puff volume, 3 s puff duration, 30 s puff interval. Measurements were then compared to those derived from the model predictions under the same conditions.

## Supplementary Information

Supplementary Information.
