# Supplementary material for: A numerical study on capillary-evaporation behavior of porous wick in electronic cigarettes
Source: Sci Rep. 2021 May 14;11:10348. doi: 10.1038/s41598-021-89685-4 (PMC8121843; doi:10.1038/s41598-021-89685-4)
Supplement: Supplementary file 1 — Supplementary Information. [file 41598_2021_89685_MOESM1_ESM.docx]

Supplementary Information of “a Numerical Study on Capillary-Evaporation Behavior of Porous Wick in Electronic Cigarettes”

Yihan Gao ^1^, Dian Li ^2^, Jiexiong Ru ^1^, Muyun Yang ^3^, Lehua Lu ^1^, Li Lu ^2^, Jinlu Wu ^3^, Zhonghui Huang ^2^, Yan Xie ^1,^* and Naiping Gao ^3,*^

^1^ Shanghai New Tobacco Product Research Institute, Xiupu Road 3733, 201315, Shanghai, China; gaoyh@sh.tobacco.com.cn (Y.G.); rujx@sh.tobacco.com.cn (J.R.); lulh@sh.tobacco.com.cn (LH.L.)

^2^ China Tobacco Guangxi Industrial Co., Ltd., Beihu South Road 28, 530001, Nanning, China; lid@gxzy.cn (D.L.); lul@gxzy.cn (L.L.); huangzh@gxzy.cn (Z.H.)

^3^ School of Mechanical Engineering, Tongji University, Caoan Highway 4800, 201804, Shanghai, China; 1830224@tongji.edu.cn (M.Y.); 1710768@tongji.edu.cn (J.W.)

***** xieyan2020@hotmail.com (Y.X.); gaonaiping@tongji.edu.cn (G.N.)

Experimental conditions

To validate the numerical model, a total of 15 experiments were carried out by varying the test parameters by the expanded-scale experimental platform (Table S1).

**Table S1**. The 15 experimental combinations under the applied power and PG/VG ratio.

| No. | PG/VG (vol%/vol%) | Electrical power of prototype  electronic cigarette (W) |
| --- | --- | --- |
| 1 | 100/0 | 1.46 |
| 2 | 100/0 | 2.40 |
| 3 | 100/0 | 3.50 |
| 4 | 100/0 | 5.32 |
| 5 | 100/0 | 8.32 |
| 6 | 50/50 | 1.36 |
| 7 | 50/50 | 2.40 |
| 8 | 50/50 | 4.56 |
| 9 | 50/50 | 6.58 |
| 10 | 50/50 | 9.09 |
| 11 | 0/100 | 1.46 |
| 12 | 0/100 | 2.50 |
| 13 | 0/100 | 4.73 |
| 14 | 0/100 | 6.64 |
| 15 | 0/100 | 8.32 |

Simulation properties and conditions

Physical properties of the prototype e-cigarette at the mean value (120℃)of the average boiling temperature (230℃)and the ambient temperature (10℃) are shown in Table S2.

**Table S2**. Physical properties of the prototype electronic cigarette. (^1^ Note: Some values are not specified because they are related to the liquid composition.)

| **Quantities** | | **Values ^1^** | **Units** |
| --- | --- | --- | --- |
| Empirical coefficients of the Antoine equation | $A$ | $3.94\sim6.08$ | ~ |
|  | $B$ | $1411\sim2693$ | ~ |
|  | $C$ | $-200.6\sim-17.9$ | ~ |
| Heat capacity of the e-liquid | $c_{p,l}$ | $2900\sim3177$ | $J kg^{-1} K^{-1}$ |
| Heat capacity of the wick | $c_{p,s}$ | $1925$ | $J kg^{-1} K^{-1}$ |
| Diameter of the wick | $D$ | $0.003$ | m |
| Diffusion coefficient in the air | $D_{i}$ | $9.13\times{10}^{-6}$~  $9.70\times{10}^{-6}$ | m |
| Heat transfer coefficient of a cylinder in cross flow | $h_{a}$ | $31.4$ | $W m^{-2} K^{-1}$ |
| Convective mass transfer coefficient | $h_{D,i}$ | $0.018$ | $m s^{-1}$ |
| Latent heat of liquid vaporization | $h_{fg}$ | $8.10\times{10}^{5}\sim$  $8.68\times{10}^{5}$ | $J kg^{-1}$ |
| Permeability of the wick | $K$ | $3.5\times{10}^{-10}\sim$  $9.5\times{10}^{-10}$ | $m s$ |
| Thermal conductivity of the cotton wick | $k_{s}$ | $0.046$ | $W m^{-1}K^{-1}$ |
| Thermal conductivity of the e-liquid | $k_{l}$ | $0.236\sim0.298$ | $W m^{-1}K^{-1}$ |
| Length of the transport section of the wick | $L_{t}$ | $0.004$ | $m$ |
| Length of the heated section of the wick | $L_{h}$ | $0.008$ | $m$ |
| Molar mass | $M_{i}$ | $76\sim92$ | $kg mol^{-1}$ |
| Ambient air temperature | $T_{0}$ | $10$ | $℃$ |
| Puff duration | $t_{a}$ | $3$ | $s$ |
| Atmospheric pressure | $P_{a}$ | $101325$ | $\mathrm{Pa}$ |
| Universal gas constant | $R$ | $8.314$ | $J mol^{-1} K^{-1}$ |
| Density of the e-liquid | $\rho_{l}$ | $933\sim1159$ | $\mathrm{kg}m^{-3}$ |
| Density of the wick | $\rho_{s}$ | 540 | $\mathrm{kg}m^{-3}$ |
| Sherwood number of evaporating mass transfer | $Sh$ | $5.83\sim5.96$ | $\sim$ |
| Schmidt number | $Sc$ | $1.53\sim1.62$ | $\sim$ |
| Surface tension coefficient | $\sigma$ | $0.036\sim0.063$ | $\mathrm{kg}s^{-2}$ |
| Dynamic viscosity of the liquid | $\mu_{l}$ | $0.34\sim5.96$ | $\mathrm{kg}m^{-1} s^{-1}$ |
| Kinematic viscosity of air | $\nu_{a}$ | $1.48\times{10}^{-5}$ | $m^{2} s^{-1}$ |

After some preliminary model validation by comparing the combined model predictions with the experimental results, a total of 64 cases which include four power levels (1.43, 3.81, 6.20, 8.58 W), four PG/VG ratios (0/100, 50/50, 75/25, 100/0 vol%/vol%) and four wick porosity levels (0.28, 0.44, 0.60, 0.76) were systematically simulated to evaluate their effects on the e-liquid temperature, vaporization rate, and thermal efficiency, as shown in Table S3.

**Table S3**. Parameters and their levels used in the simulation studies.

| **No.** | **Electrical Power (W)** | **PG/VG (vol%/vol%)** | **Wick Porosity** |
| --- | --- | --- | --- |
| 1 | 1.43 | 0/100 | 0.28 |
| 2 | 1.43 | 50/50 | 0.28 |
| 3 | 1.43 | 75/25 | 0.28 |
| 4 | 1.43 | 100/0 | 0.28 |
| 5 | 1.43 | 0/100 | 0.44 |
| 6 | 1.43 | 50/50 | 0.44 |
| 7 | 1.43 | 75/25 | 0.44 |
| 8 | 1.43 | 100/0 | 0.44 |
| 9 | 1.43 | 0/100 | 0.60 |
| 10 | 1.43 | 50/50 | 0.60 |
| 11 | 1.43 | 75/25 | 0.60 |
| 12 | 1.43 | 100/0 | 0.60 |
| 13 | 1.43 | 0/100 | 0.76 |
| 14 | 1.43 | 50/50 | 0.76 |
| 15 | 1.43 | 75/25 | 0.76 |
| 16 | 1.43 | 100/0 | 0.76 |
| 17 | 3.81 | 0/100 | 0.28 |
| 18 | 3.81 | 50/50 | 0.28 |
| 19 | 3.81 | 75/25 | 0.28 |
| 20 | 3.81 | 100/0 | 0.28 |
| 21 | 3.81 | 0/100 | 0.44 |
| 22 | 3.81 | 50/50 | 0.44 |
| 23 | 3.81 | 75/25 | 0.44 |
| 24 | 3.81 | 100/0 | 0.44 |
| 25 | 3.81 | 0/100 | 0.60 |
| 26 | 3.81 | 50/50 | 0.60 |
| 27 | 3.81 | 75/25 | 0.60 |
| 28 | 3.81 | 100/0 | 0.60 |
| 29 | 3.81 | 0/100 | 0.76 |
| 30 | 3.81 | 50/50 | 0.76 |
| 31 | 3.81 | 75/25 | 0.76 |
| 32 | 3.81 | 100/0 | 0.76 |
| 33 | 6.20 | 0/100 | 0.28 |
| 34 | 6.20 | 50/50 | 0.28 |
| 35 | 6.20 | 75/25 | 0.28 |
| 36 | 6.20 | 100/0 | 0.28 |
| 37 | 6.20 | 0/100 | 0.44 |
| 38 | 6.20 | 50/50 | 0.44 |
| 39 | 6.20 | 75/25 | 0.44 |
| 40 | 6.20 | 100/0 | 0.44 |
| 41 | 6.20 | 0/100 | 0.60 |
| 42 | 6.20 | 50/50 | 0.60 |
| 43 | 6.20 | 75/25 | 0.60 |
| 44 | 6.20 | 100/0 | 0.60 |
| 45 | 6.20 | 0/100 | 0.76 |
| 46 | 6.20 | 50/50 | 0.76 |
| 47 | 6.20 | 75/25 | 0.76 |
| 48 | 6.20 | 100/0 | 0.76 |
| 49 | 8.58 | 0/100 | 0.28 |
| 50 | 8.58 | 50/50 | 0.28 |
| 51 | 8.58 | 75/25 | 0.28 |
| 52 | 8.58 | 100/0 | 0.28 |
| 53 | 8.58 | 0/100 | 0.44 |
| 54 | 8.58 | 50/50 | 0.44 |
| 55 | 8.58 | 75/25 | 0.44 |
| 56 | 8.58 | 100/0 | 0.44 |
| 57 | 8.58 | 0/100 | 0.60 |
| 58 | 8.58 | 50/50 | 0.60 |
| 59 | 8.58 | 75/25 | 0.60 |
| 60 | 8.58 | 100/0 | 0.60 |
| 61 | 8.58 | 0/100 | 0.76 |
| 62 | 8.58 | 50/50 | 0.76 |
| 63 | 8.58 | 75/25 | 0.76 |
| 64 | 8.58 | 100/0 | 0.76 |
